# Measuring the dynamic response of the thylakoid architecture in plant leaves by electron microscopy

**DOI:** 10.1002/pld3.280

**Published:** 2020-11-05

**Authors:** Meng Li, Roma Mukhopadhyay, Václav Svoboda, Hui Min Olivia Oung, Daniel L. Mullendore, Helmut Kirchhoff

**Affiliations:** ^1^ Institute of Biological Chemistry Washington State University Pullman WA USA; ^2^ Franceschi Microscopy and Imaging Center Washington State University Pullman WA USA; ^3^ Present address: School of Oceanography University of Washington Seattle WA USA

**Keywords:** Arabidopsis thaliana, high‐pressure freezing, photosynthesis, stromal gap, thylakoid lumen, thylakoid membrane, transmission electron microscopy

## Abstract

The performance of the photosynthesis machinery in plants, including light harvesting, electron transport, and protein repair, is controlled by structural changes in the thylakoid membrane system inside the chloroplasts. In particular, the structure of the stacked grana area of thylakoid membranes is highly dynamic, changing in response to different environmental cues such as light intensity. For example, the aqueous thylakoid lumen enclosed by thylakoid membranes in grana has been documented to swell in the presence of light. However, light‐induced alteration of the stromal gap in the stacked grana (partition gap) and of the unstacked stroma lamellae has not been well characterized. Light‐induced changes in the entire thylakoid membrane system, including the lumen in both stacked and unstacked domains as well as the partition gap, are presented here, and the functional implications are discussed. This structural analysis was made possible by development of a robust semi‐automated image analysis method combined with optimized plant tissue fixation techniques for transmission electron microscopy generating quantitative structural results for the analysis of thylakoid ultrastructure.

**Significance Statement:**

A methodical pipeline ranging from optimized leaf tissue preparation for electron microscopy to quantitative image analysis was established. This methodical development was employed to study details of light‐induced changes in the plant thylakoid ultrastructure. It was found that the lumen of the entire thylakoid system (stacked and unstacked domains) undergoes light‐induced swelling, whereas adjacent membranes on the stroma side in stacked grana thylakoid approach each other.

## 1 INTRODUCTION

In plants, the conversion of sunlight into metabolic energy equivalents by photosynthetic light reactions takes place in the thylakoid membrane system inside the chloroplast. The chloroplast space is partitioned by this membrane into two aqueous sub‐compartments, the thylakoid lumen and the stroma. The thylakoid membrane is composed of the compact cylindrical grana (300–600 nm in diameter) made of stacked membranes and the unstacked stroma lamellae that interconnects the grana stacks (Bussi et al., 2019; Dekker & Boekema, 2005). It has been hypothesized that in higher plants, grana stacks have been selected during evolution for fine‐tuning and optimizing energy conversion in particular under fluctuating light conditions (Anderson et al., 2012; Chow et al., 2005; Horton, 1999; Mullineaux, 2005). The energy‐converting multi‐protein complexes that are embedded in thylakoid membranes enable light harvesting (light‐harvesting complexes, LHC), electron transfer (photosystem (PS)I, PSII, cytochrome *b_6_f* complex) and ATP synthesis (ATP synthase complex). In addition, a number of low‐abundant proteins are involved in regulation of light harvesting and electron transport (e.g., non‐photochemical quenching, state transitions, cyclic electron transport) and maintenance of those functions (e.g., PSII repair). It was suggested that light‐induced changes in the thylakoid ultrastructure is a mean to facilitate and control protein mobility that is compromised by macromolecular crowding in thylakoid membranes (Kaňa, 2013; Kirchhoff, 2014a; Kirchhoff et al., 2011; Wood et al., 2018). For example, only about 50% of the protein complexes are mobile in the unstacked thylakoid membranes and even less than 20% in the stacked grana regions (Kirchhoff et al., 2013; Murphy, 1986). The different protein mobilities can be explained by the lower and higher protein‐packing densities in unstacked and stacked thylakoid regions, respectively (Kirchhoff et al., 2013). A well‐established example for thylakoid membrane dynamics is that light intensity controls the number of stacked membranes in grana, that is, low light‐adapted plants exhibit more membranes per grana stack compared to their high light‐adapted counterparts (Anderson et al., 2012). In addition to light‐induced changes in the number of membranes per grana, the lateral dimension (diameter) of the grana cylinder is variable (Anderson et al., 2012; Chuartzman et al., 2008; Herbstová et al., 2012; Kirchhoff, 2019; Wood et al., 2019). Furthermore, evidence exists that the vertical dimension (width) of the lumen in stacked grana can vary between dark‐ and light‐adapted plants. This variation has been studied earlier by monitoring changes of repeat distance in grana (Kirchhoff et al., 2011; Murakami & Packer, 1970). The repeat distance may be defined as the vertical distance from the middle of one stromal gap in a grana stack to the middle of the neighbored stromal gap. Previous studies have illustrated that dark‐adapted plants have a reduced repeat distance compared to light‐adapted plants (Kirchhoff et al., 2011). However, the exact changes in the distances of the lumen and stromal gap are often difficult to obtain due to sample preparation and imaging issues.

Recent breakthroughs in electron tomography have provided unprecedented insights into the three‐dimension organization of thylakoid membranes (Austin & Staehelin, 2011; Bussi et al., 2019; Daum et al., 2010; Mustárdy et al., 2008; Shimoni et al., 2005). However, tomography preparation, reconstruction, and analysis are labor intensive. Moreover, because tomography is time consuming, it may not be possible to acquire large amounts of data from multiple datasets and conditions to perform adequate statistical analysis of chloroplast ultrastructure. Classical transmission electron microscopy (TEM) on ultrathin‐sectioned material is a preferred method to study thylakoid membrane organization within leaf tissues to collect statistically relevant results. Because sample preparation techniques for TEM are prone to generating artifacts, it is paramount to optimize these sample preparations and evaluate samples for these potential artifacts. High‐pressure freezing (HPF) followed by freeze substitution (together HPF‐FS) has been developed as a fixation method to preserve the “native state” for microbial samples, animal tissues, and plant tissues (Charuvi et al., 2016; Hess, 2007). Until now some studies used higher plant leaf tissue for studying the thylakoid ultrastructure by HPF‐FS and TEM (e.g., Bourett et al., 1999; Bussi et al., 2019; Charuvi et al., 2016; Pfeiffer & Krupinska, 2005). Yet, the quality assessment of those images often was not critically discussed. In this work, we explored different leaf tissue preparation conditions for HPF and report a repeatable method of preserving the “near‐native” state of leaf tissue and thylakoid structure, with minimal artifacts. This is also compared with microwave‐assisted chemical fixation. To assess the quality of samples and images obtained using HPF‐FS and TEM, we present a hierarchical image quality control procedure to select the best samples for quantitative analysis. The optimized TEM protocols were complemented by new image analysis tools that provide unbiased quantification of key structural parameters of thylakoid membranes. These TEM‐based toolsets will be useful for future work on quantitative analyses of the thylakoid ultrastructure derived from leaf tissues allowing better understanding of environmental‐triggered changes in photosynthetic membranes.

## 2 MATERIAL AND METHODS

### 2.1 Plant material

Experiments were performed with 5‐ to 7‐week‐old *Arabidopsis thaliana* plants maintained in growth chamber with constant temperature at 21°C, and 9 hr illumination per day at 120 µmol/m^2^/s. Before sample preparation, plants were either dark‐adapted overnight or illuminated with 250–300 µmol/m^2^/s for 30 min.

### 2.2 High‐pressure freezing‐freeze substituted (HPF‐FS) fixation

HPF carriers, B type (Leica) were precoated with lecithin or 1‐hexadecene before use. Before assembling the HPF carriers for freezing, *Arabidopsis* leaf discs were harvested using a leaf punch, on a dental wax plate preloaded with droplets of cryoprotectant solutions. The leaf discs were either pre‐infiltrated with cryoprotectant using a syringe before detaching the dark/light–adapted leaf from the plant, or vacuum infiltrated with cryoprotectant after leaf discs were taken from leaves. In the carriers and outside the leaf discs, the cryo‐filler, or external cryoprotectant fillers, was either the same as the cryoprotectant or different in a few cases. After testing a few cryoprotectants and the two methods of infiltration, we noticed that vacuum infiltration was rarely successful in preserving the leaf structures after HPF (Table S1). Therefore, for the following experiments, we used the pre‐infiltration method. Leaf discs samples loaded in HPF carriers were frozen at a rate of approximately 20,000 K/s, using the high‐pressure freezing machine HPM100 (Leica). After HPF, the discs were transferred into freeze substitution (FS) fixatives frozen and stored in liquid N_2_. Initially, the fixative containing 1% glutaraldehyde, 1% OsO_4_, 1% ZnI_2_, and 2.5% H_2_O in acetone was used similar as described by Koochak et al. (Koochak et al., 2019). Later the fixative was modified (0.5% OsO_4_, 0.25% glutaraldehyde, and 0.2% uranyl acetate in acetone) to improve visibility after FS, which was used in all successful HPF‐FS preparation in this study. The FS and resin infiltration steps were as described by Koochak et al. (Koochak et al., 2019), while Spurr's resin was in this study.

### 2.3 Microwave fixation

The protocol is based on the method described by Zechmann and Zellnig (Zechmann & Zellnig, 2009) as detailed in the following. Leaf discs around 2 mm in diameter were punched out and fixed with two different buffers.

*Buffer 1*: Leave disks were placed in 4% glutaraldehyde, 2 mM calcium chloride, and 25 mM cacodylate buffer, pH 7.2. The samples were microwave fixed on ice two times for 1 min each at 350 watts with temperature restriction set at 32°C, with 1‐min break in between microwave cycles. The samples were rinsed three times, 10 min each in 50 mM cacodylate buffer, pH 7.2. The samples were postfixed overnight at 4ºC in reduced osmium (1.5% potassium ferrocyanide, 50 mM cacodylate (pH 7.2), 2 mM calcium chloride, and 2% osmium tetroxide) to chelate osmium intermediates into the lumen of the endomembrane systems (Carde, 1987; White et al., 1979). The samples were rinsed three times in water for 10 min each step before acetone microwave dehydration (below).

*Buffer 2*: Leave disks were placed in 2% paraformaldehyde, 2% glutaraldehyde, and 50 mM cacodylate, pH 7.2. The samples were microwave fixed on ice 3 times, 1 min each at 750 watts with a restriction temperature set at 28°C, with a 5‐min rest in between microwave cycles. The samples were rinsed three times, 5 min each in 50 mM cacodylate buffer, pH 7.2. The samples were postfixed for 1 hr at 25ºC in 2% OsO_4_ in 25 mM cacodylate buffer, pH 7.2. The samples were rinsed three times in water for 10 min each step before ethanol microwave dehydration.

### 2.4 Dehydration

Microwave dehydration was performed in a graded acetone (Buffer 1) or ethanol series (Buffer 2) as follows: 30%, 50%, 60%, 70%, 80%, 90%, 95%, and 100% (three times) at 750 watts, for 1 min each step with a restriction temperature of 35°C. The dehydrating solvent was replaced with the transition solvent propylene oxide at 1:1 solvent: propylene oxide ratio for 10 min at RT, followed by 100% propylene oxide (two times) for 10 min at RT. The samples were infiltrated slowly with Spurr's resin and propylene oxide as follows: Spurr's:propylene oxide—1:3 ratio (25%), 1:2 (33%), 1:1 (50%), 2:1 (66%), 3:1 (75%), and 100% Spurr's (three times), each infiltration step was allowed to infiltrate overnight. The resin was exchanged just prior to embedding in molds and polymerized overnight at 70°C.

### 2.5 Transmission electron microscopy (TEM)

Samples from high‐pressure fixation and microwave fixation were thin sectioning, stained, and imaged as described in Froelich et al. (Froelich et al., 2011). In short, thin sections of 70–100 nm were taken with a Reichert Ultracut R Ultramicrotome, collected on formvar‐coated slot grids, poststained for 20 min in 2% aqueous uranyl acetate, and 8 min in Reynold's lead and imaged with a ThermoScientific Tecnai F20 microscope. Thick sections of 500–1,000 nm were collected for light microscopy check.

### 2.6 Image processing

**FIGURE 1:**
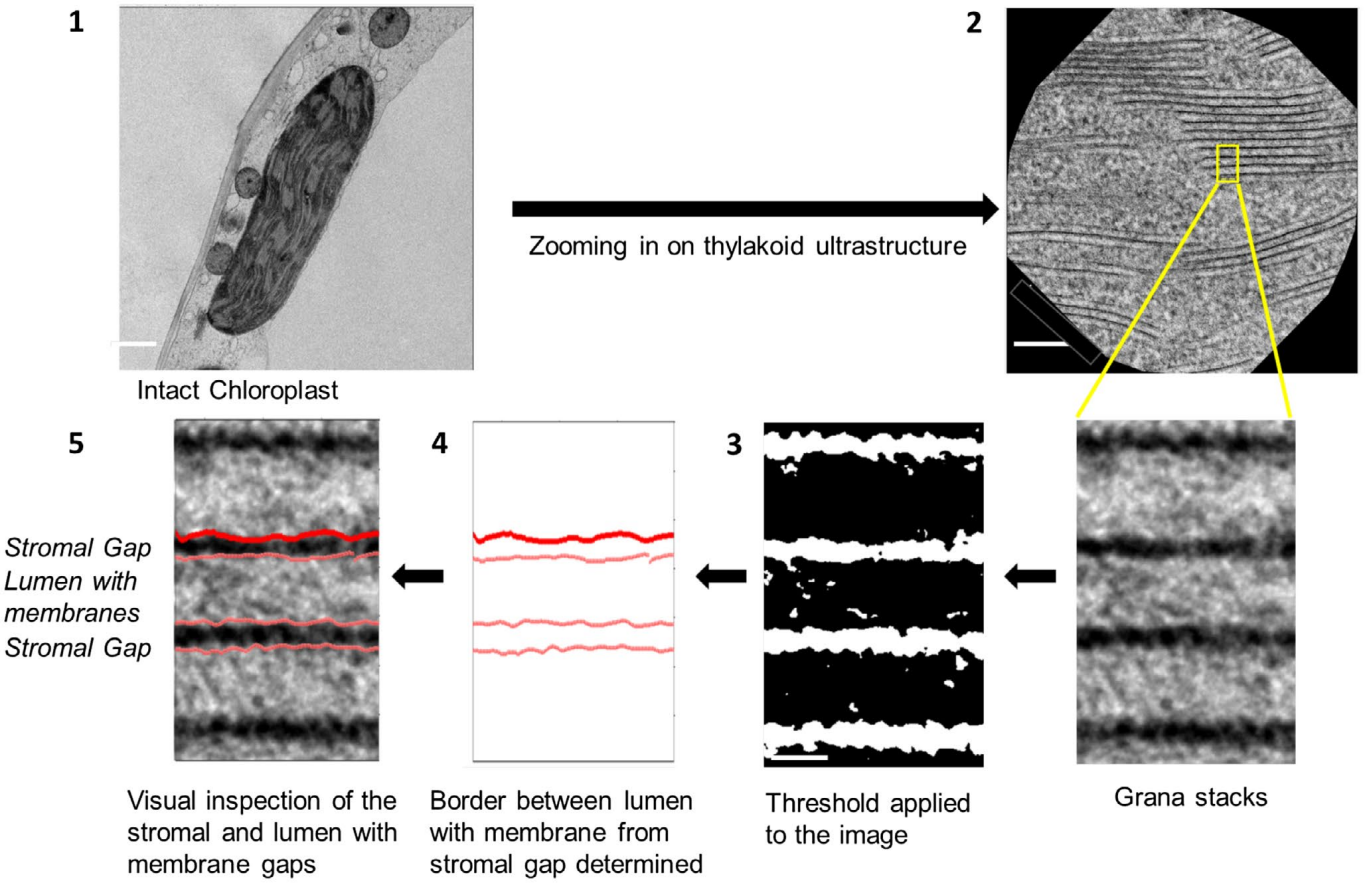
Schematic diagram of the methodology to analyze the TEM images. 1. An intact chloroplast from HPF‐treated leaf tissue is selected; 2. The thylakoid structure of the chloroplast is zoomed in; 3. The image is then converted into a black and white image; 4. Border of stripes are extracted, and borders are plotted; 5. The graph is overlapped to the original image for visual inspection. Scale bars: (1) 1µm, (2) 100 nm, and (3) 5 nm

**FIGURE 2:**
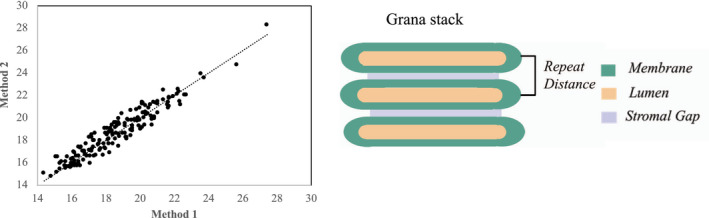
Correlation between Method 1 and Method 2 for calculation of the repeat distance in grana stacks (shown as a cartoon diagram on the right). See in the text for details

The image processing was done using ImageJ software (Rueden et al., 2017). The TEM images were selected based on the structural intactness of tissue, intactness of cell, absence of plasmolysis, and intactness of chloroplasts (see section *Quality check for TEM images from HPF samples*). The details of the two methods (Method 1 and Method 2) are described in detail in Supplementary Information. The image analysis procedure for Method 2 is summarized in Figure 1. The repeat distance in stacked grana was calculated by both methods and plotted against each other (Figure 2). We employed the two methods for estimating the repeat distance because Method 1 (Figure S1) function as a quality control of Method 2, that is, the sum of stroma gap and lumen plus membranes calculated by Method 2 should give the repeat distance determined by Method 1. The reason for deviations between the two methods seen in Figure 2 is either due to poor fit of the sinusoidal curve to the grana image from Method 1 or low contrast in TEM images making evaluation of the stromal gap by Method 2 challenging. We used the cross‐comparison between both methods as the criterion for the image analysis, that is, only if both methods give repeat distances that deviate by less than 9% (set as an arbitrary threshold), the image analysis is accepted. By adding this constriction, we can evaluate the lumen and stromal gap with high confidence. Overall, both methods give very similar repeat distances indicated by the high correlation coefficient (Figure 2). The statistical *t*‐test (Mann‐Whitney Rank‐Sum test) for the data including graphical plots was done using SigmaPlot ® 11.0 software (Systat, 2008).

## 3 RESULTS

### 3.1 Quality check of TEM images

**FIGURE 3:**
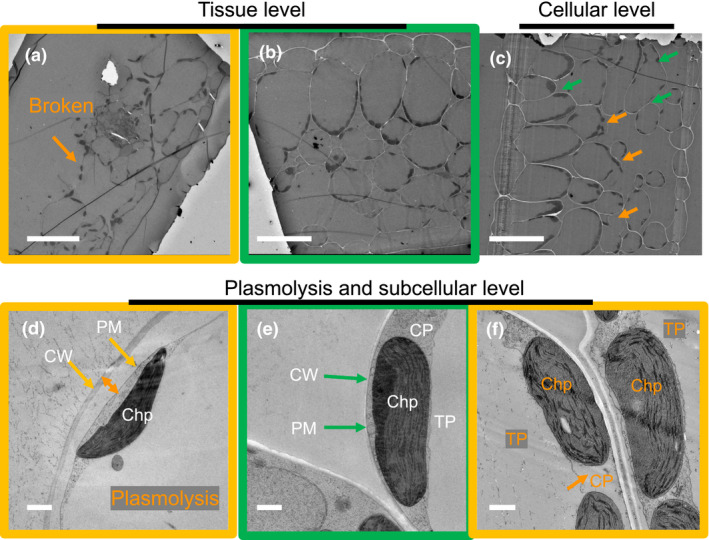
Quality check for TEM images from HPF‐FS samples: The images summarize our hierarchical procedure (from top left to bottom right) to evaluate the quality of EM images ranging from the tissue, to the cellular and subcellular level. The following structures are denoted: cell wall (CW), plasma membrane (PM), cytoplasm (CP), chloroplast (Chp), and tonoplast (TP). Green arrows and boxes indicate structures that pass the quality check; orange labels and boxes denote damaged structures with reasons and locations colored. Scale bars: upper panels, 50 µm; lower panels, 1 µm

An inevitable consequence of sample preparation for electron microscopy is that artificial ultrastructural alterations of the native structure occur due to mechanical or chemical impacts. To evaluate the quality of the HPF‐FS fixation of a leaf sample, the preservation of leaf tissue, cellular and sub‐cellular (organelle) structures were sequentially assessed as summarized in Figure 3. Before cellular and sub‐cellular inspection, the samples can be screened at the leaf tissue level using light microscopy after staining thick sections (500–1,000 nm) from the sample block. After leaf tissue level screening, cellular and sub‐cellular structures were inspected with TEM imaging. This quality check can also be done by a live inspection of images directly at the electron microscope stage without taking unnecessary microscopy time. Only for those images that pass all‐level test criteria, a detailed ultrastructural analysis of the thylakoid membranes was conducted (below). At the leaf tissue level, many leaf samples after HPF‐FS were broken (Table S1, Figure 3). A very common artifact is the leaf disc usually broke at the central plane of the disc and divide the leaf into two halves (Figure 3a shows one half), analogous to “pita bread” whose center is hollow. At the cellular level, the overall structure of cells was assessed by checking the presence of ice/mechanical damage on the cell wall. Damaged cells usually appear as exploded, as indicated in Figure 3c (orange arrows). Plasmolysis (Figure 3d) usually can be found when the cryoprotectant solution is hypertonic (Table S1), which can be avoided by lowering the concentration of cryoprotectant. At the sub‐cellular level, the integrity of cytoplasm, tonoplast, chloroplasts, and other organelles was inspected. While a high‐quality fixation leads to smooth cytoplasm membranes, tonoplast membranes (Figure 3e), and relatively homogeneous cytoplasm altogether, a poor preservation usually shows the opposite with disrupted tonoplast membrane and discontinuous cytoplasm (Figure 3f, Figure S2a,b). Besides the general criteria discussed above, the preservation of other organelles, including, but not limited to, Golgi apparatus, nucleus, and microtubules (Figure S2c–e,h), is also used to facilitate the selection of quality samples and images. In some cases, even when the chloroplast seemed well preserved, artifacts persist as compressed thylakoids (grana stacks), which usually appear as heavily stained areas (Figure S2e,f). The thylakoid layers in those areas are either too dark to distinguish each layer or appear extremely compressed (with repeat distance often <13 nm) (Figure S2f,g), beyond the theoretical threshold of accommodating a PSII with a protrusion to the lumen (Daum et al., 2010; Kirchhoff et al., 2011). Even though there are multiple aspects to inspect the quality assessment process, it turns out that this is a straightforward procedure that does not require sophisticated image analysis tools. This image quality check increases the likelihood that grana will be analyzed with well‐preserved ultrastructure.

### 3.2 Testing cryoprotectants for HPF treatment

For high‐pressure freezing, the quality of sample preservation is dependent on the fast vitrification of water within leaf tissue. Water is a natural thermal insulator, whereas plant cells can be up to 90% water are prone to freezing artifacts, therefore, cryoprotectants are employed to circumvent the natural properties of water to produce damaging ice crystals during a freezing event (Gilkey & Staehelin, 1986; Kiss et al., 1990). However, the intrinsic air space within an *Arabidopsis* leaf prevents the preservation of leaf structure even with cryoprotectant outside the leaf tissue. Without the air space filled by a cryoprotectant, the air inside the leaf would contract during HPF leading to potential collapse of leaf tissue and later explode when temperature slowly rises to room temperature. As a result, researchers have used vacuum to infiltrate the leaf tissue to fill the air space with cryoprotectants, or directly used 1‐hexadecene to remove air within a leaf tissue (Austin, 2014). With limitation on extracellular cryoprotectant components to nonpolar chemicals, and the concern that nonpolar solvent could innately introduce some artifacts to the leaf tissue and membrane structure, we did not attempt to use 1‐hexadecene as a cryoprotectant. Table S1 surveys the impact of different cryoprotectants, cryo‐fillers, and infiltration methods on the preservation quality of HPF‐FS fixed leaf tissue samples. Initially, we tried published methods with vacuum infiltration, yet to our surprise, none of the sample preparations yielded a whole leaf tissue structure (Table S1). Considering low efficiency of vacuum infiltration, we applied syringe infiltration by gently pushing cryoprotectants into leaves. After noticing encouraging signs from leaf tissues infiltrated with 0.33 M sorbitol and yeast paste as cryo‐filler (Table S1), the cryoprotectant and cryo‐filler were optimized and led to our first observation of intact leaf tissue structures in 300 mM sorbitol infiltrated leaf samples (Table S1). The result of leaf samples infiltrated with 300 mM sorbitol in 10% methanol and using the same cryo‐filler was most promising, which later showed relative consistent performance in preserving whole leaf disc structure as well as cellular and subcellular features. We tried to remove or reduce methanol from our cryoprotectant/cryo‐filler, but with limited experiments, no comparable efficiency or performance was achieved (Table S1).

Quantifications of the grana vertical dimension from sample images that passed the quality check reveal that samples infiltrated using the cryoprotectant 300 mM sorbitol with 10% methanol show reproducible repeat distances (Table S2). Furthermore, omitting methanol did not lead to significant alterations of the ultrastructure (Table S2). This indicates that the addition of 10% methanol, while helping to preserve overall tissue to cellular structures of leaf sample during HPF, introduced little, if any, artifact to thylakoid ultrastructure. In contrast, anaerobic condition in combination with NaHCO_3_ addition appears to have compressed thylakoids with average repeat distance ~15.3 nm (Table S2). For our optimized preparation conditions (300 mM sorbitol with 10% methanol) both methods 1 and 2 give very similar repeat distances for dark‐ and light‐adapted samples (Table 1). The stroma gaps are slightly (~15%) smaller for method 1 compared to 2 (Table 1). This is caused by the fact that method 1 estimates the stroma gap from the full width half maximum (FWHM) of a curve fit of the stroma gap (Figure S1), whereas in method 2 this number is extracted from a rectangular‐shaped representation of the stroma gap (Figure 1). Since method 2 provides a clear (steep) border between stroma gap and membranes, we will employ this method in the following for ultrastructural characterizations. The stacking repeat distances of 17.0 ± 0.2 nm is consistent with previous studies on cryoimmobilized *Arabidopsis* leaf samples (16.3 to 16.8 nm, (Kirchhoff et al., 2011)).

**Table 1:** Analyses of TEM images from HPF‐FS leaf samples reveal light‐induced changes in thylakoid architecture

Leaf treatment	Resin curing date	Cryoprotectant	No. of leaf discs (sample blocks) analyzed	Method 1				Method 2			
				Sample size^a^ (layers of thylakoid repeats)	Lumen + membranes (nm, mean ± SEM)	Stroma gap (nm, mean ± SEM)	Repeat distance (nm, mean ± SEM)	Sample size	Lumen + membranes (nm, mean ± SEM)	Stroma gap (nm, mean ± SEM)	Repeat distance (nm, mean ± SEM)
Dark adapted	Jan‐2019	300 mM Sorbitol + 10% methanol	2	1,080	13.21 ± 0.06	3.90 ± 0.03	17.09 ± 0.06	113	12.22 ± 0.21	4.68 ± 0.10	16.98 ± 0.17
Light adapted	Jan‐2019	300 mM Sorbitol + 10% methanol	2	1,080	16.33 ± 0.10	3.55 ± 0.03	19.89 ± 0.10	252	15.68 ± 0.19	4.20 ± 0.07	20.04 ± 0.17

^a^ Sample size is the number of Repeat distances analyzed; in general: *N*(stromal gap) > *N*(Repeat distance) > *N*(Lumen + membranes).

### 3.3 Light‐induced alterations of the grana thylakoid ultrastructure

**FIGURE 4:**
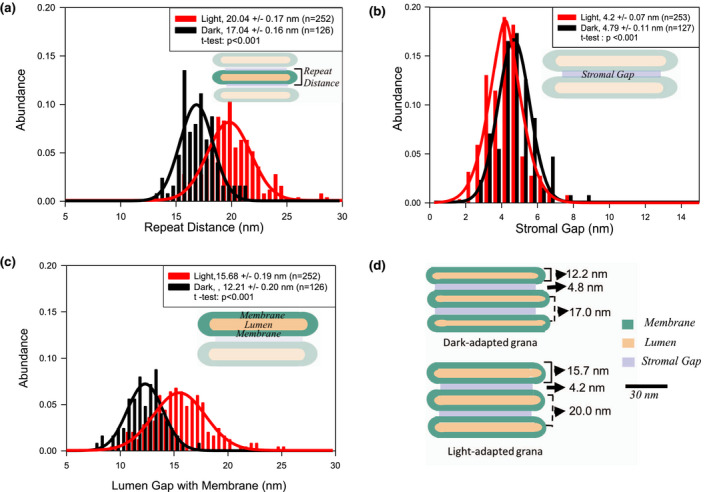
Change in thylakoid architecture in dark‐ and light‐adapted plants after HPF fixation. A. Difference in repeat distance, B. Difference in the stromal gap, C. Difference in the lumen, and D. In‐scale model (in vertical dimension) of grana in dark‐ and light‐adapted thylakoids summarizing the data in A to C. *R* ^2^ values for the Gaussian fittings for dark/light–adapted samples are .92/.94 (Figure 4a), .86/.93 (b), and .94/.93 (c)

To investigate if the HPF method can capture the light‐induced thylakoid ultrastructural changes, the analysis was performed for dark‐ and light‐adapted leaf samples that were harvested and processed through HPF‐FS in the same experiment from the same day (Table 1). The results of this analysis are summarized in the histograms in Figure 4. The repeat distance between two adjacent grana discs (measured from the middle of one stroma gap to the middle of the neighbored one) increased from 17.0 to 20.0 nm in light‐adapted compared to dark‐adapted samples (Figure 4a). This light‐induced widening of the vertical grana structure is accompanied by a slight but significant 13% decrease of ~0.6 nm for the stromal gap in the light‐adapted plants (Figure 4b), that is, adjacent grana membranes get closer together on the stroma side as was observed earlier (Kirchhoff et al., 2017). In contrast, the thickness of the whitish stripe in HPF samples (see, e.g., Figure 1) representing two membranes enclosing one lumen gets wider by ~3.5 nm. Since the membrane thickness shrinks slightly in the light (see below), this widening in light‐adapted plants must be caused by swelling of the thylakoid lumen. The difference in repeat distance caused by swelling of the thylakoid lumen in light‐adapted plants is consistent with the previously published result (Kirchhoff et al., 2011). All light‐induced structural changes are summarized by the (vertically) scaled model in Figure 4d. The values for the lipid bilayer membrane cannot be obtained from HPF images since they form only one low‐contrast strip with two membranes that are indistinguishable from the lumen (see Figure 1).

### 3.4 Microwave fixation versus HPF

**FIGURE 5:**
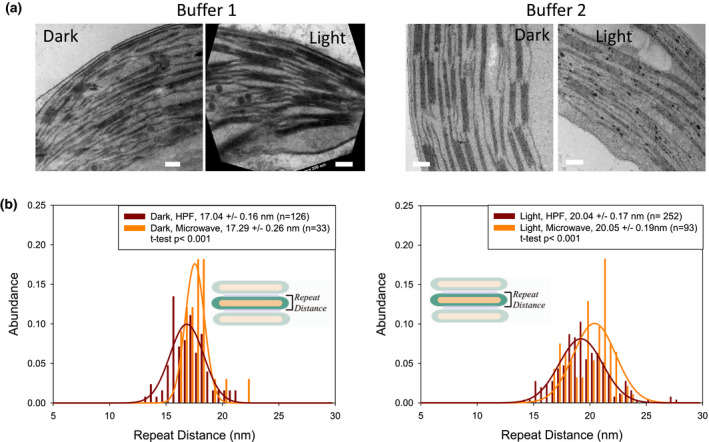
Microwave‐fixed samples. A. Examples of TEM images for dark‐ and light‐treated leaf discs fixed with buffer 1 (left) or buffer 2 (right). Scale bars: 200 nm. B. The difference in repeat distance analysis of microwave‐fixed (buffer 1) versus HPF method for dark‐ and light‐adapted conditions

It was reported that the microwave fixation procedure preserves the leaf ultrastructure similarly well as HPF or other cryo‐techniques (Kuo, 2014; Zechmann & Zellnig, 2009). We tested two different buffer systems for microwave fixation (see methods). In accordance with the literature (Carde, 1987; White et al., 1979), the image contrast is stronger with buffer 1 due to the ferrocyanide‐reduced osmium chelation compared to just the osmiophilic lipid reaction of buffer 2 (Figure 5a). This enhanced contrast helps with the computer‐based analysis of ultrastructural thylakoid parameters. We, therefore, used buffer 1 for comparison with HPF fixed samples. Ultrastructural thylakoid parameters determined for both buffer systems used for microwave fixation are summarized in Figure S3. From this comparison, it is apparent that the repeat distance for microwave fixed samples is dependent on postfixation buffer conditions. Therefore, as for HPF sample preparation, an optimized buffer system is critical for getting reliable information about the thylakoid ultrastructure for microwave fixed samples. Interestingly the microwave fixation method produces darker stained membranes that allow distinction among membranes, stroma gap, and lumen (see Figure 7 for examples), that is, the membrane thickness can be studied on microwave‐fixed samples (see below). To test whether attributes of the thylakoid ultrastructure are preserved by this technique images from HPF and microwave‐fixed leaf tissues were compared. The analysis in Figure 5 reveals that repeat distances for dark‐ and light‐treated samples is very similar between HPF and microwave‐fixed samples.

### 3.5 Ultrastructural dynamics of stroma lamellae and thylakoid membrane thickness

**FIGURE 6:**
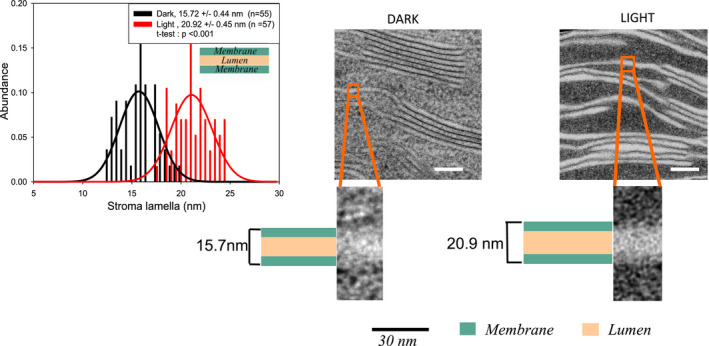
Thickness of stroma lamellae from HPF fixed dark‐ and light‐adapted plants show variation. Scale bars: 100 nm. *R* ^2^ values for the Gaussian fittings for dark/light–adapted samples are .65/.42

**FIGURE 7:**
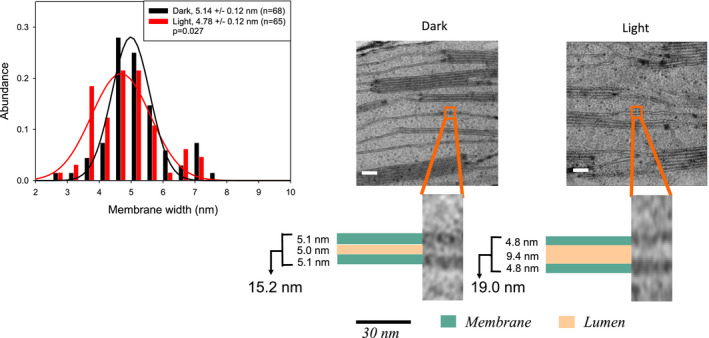
Light‐induced changes in thylakoid stroma lamella structure (right) and membrane thickness (histogram above) from microwave‐fixed samples. Scale bars: 30 nm. *R* ^2^ values for the Gaussian fittings for dark/light–adapted samples are .94/.90

So far, ultrastructural studies on thylakoid membranes focused mainly on the organization of stacked grana. Information on light‐induced changes in unstacked stroma lamellae is missing. From HPF‐treated samples, the thickness of single stroma lamellae (including two membranes enclosing a lumen) increased from 15.7 nm in dark‐adapted leaf discs to 20.9 nm in the light (Figure 6), that is, they show a similar swelling as was seen for the stacked counterparts (Figure 4a). It is noteworthy that the dimension for the membrane‐lumen‐membrane system seen in HPF prepared samples is ~30% wider in unstacked compared to stacked thylakoids for both dark‐ (15.7 vs. 12.2 nm) and light‐ (20.9 vs. 15.7 nm) adapted leaves. A similar but slight lesser swelling of stroma membrane‐lumen‐membrane system is apparent for microwave‐fixed samples as well (Figure 7). As mentioned above, due to different contrast, the microwave‐fixed samples offer the possibility to examine changes in membrane thickness and lumenal width separately. The width of the lumen expands by illumination from 5.0 nm to 9.4 nm in stroma lamellae (Figure 7, right). Single membranes as analyzed from the stroma lamellae of microwave‐fixed samples show that, for light‐adapted samples, the membrane size is statistically significantly smaller (4.8 ± 0.1 nm) than that for the dark‐adapted plant tissue (5.1 ± 0.1 nm), that is, they shrink by ~7% (Figure 7). Although microwave‐fixed samples provide more information regarding membrane thickness and lumenal width, the overall contrast of HPF‐fixed samples is better than that of microwave‐fixed samples (compare Figures 6 and 7). The sharpness in image contrast for HPF‐fixed samples have been previously reported by Pfeiffer and Krupinska (Pfeiffer & Krupinska, 2005).

## 4 DISCUSSION

In this study, optimized conditions were identified for HPF on leaf discs for studying the ultrastructural dynamics of plant thylakoid membranes. Furthermore, the TEM quality check provides a simple tool to evaluate and select images based on the structural integrity of the sample. It has to be mentioned that although HPF gives high‐quality samples for TEM analysis, the success rate of this method is low. In our hands only 1 of 7 samples prepared using the repeatable HPF‐FS method pass our quality check, that is, HPF‐TEM is time consuming. Microwave fixation of plant tissue is a much faster alternative and experimentally easier with the additional benefit that thylakoid membranes can be resolved and discriminated from the stroma gap and thylakoid lumen in contrast to HPF. We applied microwave fixation successfully for ferns and the resurrection plant *Craterostigma pumilum*. However, small tweaks in the fixation and buffer concentrations might be required to optimize this method for other plant leaves. Conventional chemical fixation method may not preserve intact cellular structure (Kiss et al., 1990) and was, thus, not used in these studies. The quantitative comparison between microwave‐ and HPF‐fixed plant tissues revealed that this is a viable alternative. However, as for HPF, proper buffer conditions are key for employing microwave fixation of plant tissues.

**FIGURE 8:**
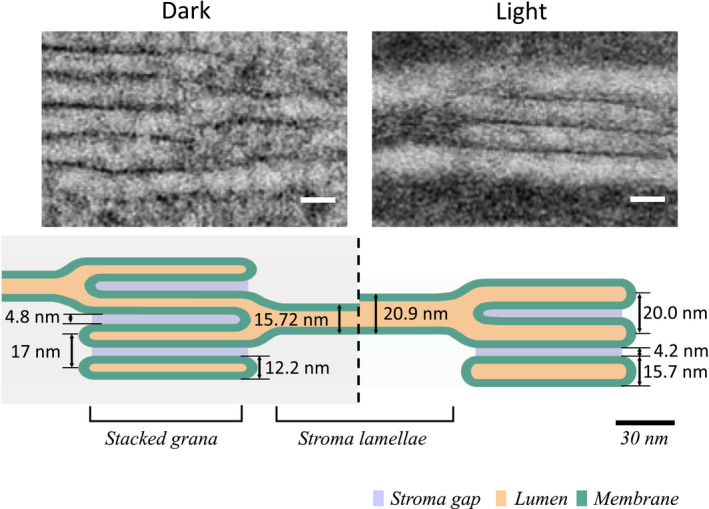
In‐scale diagram of the change in the thylakoid architecture in the presence and absence of light. Scale bars: 15 nm

The light‐induced changes in the thylakoid ultrastructure including both stacked and unstacked domains are summarized in Figure 8. Overall, the numbers derived here are in good agreement with electron tomographic analysis of the thylakoid architecture (Daum et al., 2010; Kirchhoff et al., 2011; Wietrzynski et al., 2020). Figure 8 reveals that light triggers a swelling of the lumen in the entire thylakoid system (grana and stroma lamellae). The ~30% narrower lumen in stacked compared to unstacked thylakoid regions might indicate that either attractive forces between lumenal proteins in grana (i.e., by PSII protrusions) lead to an approaching of two membranes as was postulated earlier (Albertsson, 1982, 2001) or that bending forces in grana margins mediated by structural proteins like the recently discovered *Curvature Thylakoid 1* proteins (CURT1, (Armbruster et al., 2013)) or the *Reduced Induction of Quenching* (RIQ) proteins (Yokoyama et al., 2016) squeeze two grana membranes together. Overall, this result agrees with previous studies on stacked grana showing a light‐induced swelling of the lumen in grana (Kirchhoff et al., 2011). It should be mentioned that light‐induced swelling of the thylakoid lumen is discussed controversially in the literature. For example, neutron scattering analysis for the grana repeat distance in plants give typically larger values and do not show light‐induced swelling of the grana lumen (Ünnep et al., 2014). The reason for this discrepancy is unclear as discussed in Ünnep et al. (2014). However, a recent study where neutron scattering data were rigorously modeled revealed that the first Bragg peak (used to measure the grana repeat) is not a simple measure of the grana repeat distance (Jakubauskas et al., 2019).

The dynamic change in the thylakoid architecture reported here is expected to have functional consequences. For example, swelling of the thylakoid lumen could facilitate the diffusion of small proteins, such as plastocyanin (PC), which has a molecular weight of 10.5 kDa protein (dimension of ~4 × 3 × 3 nm (Guss et al., 1986)). Plastocyanin is involved in transferring electrons from cytochrome *b_6_f* (cyt *b_6_f*) complex to PSI. The swelling of the lumen was postulated to increase the mobility of PC in the protein crowded lumen space in the grana (Kirchhoff et al., 2011). Besides PC, the mobility and/or sublocalization of other lumen‐hosted proteins involved in photoprotection (i.e., *violaxanthin de‐epoxidase*, VDE) or protein repair (i.e., DEG proteases) is expected to be influenced by lumenal swelling and shrinkage (Kirchhoff, 2013, 2014b). Furthermore, small changes in the stroma gap may influence the sublocalization of the cyt *b_6_f* complex and other membrane proteins with stromal protrusions in thylakoid membranes. In detail, the stromal side of the dimeric cyt *b_6_f* complex has a significant protrusion of cyt *f* subunit as observed in high‐resolution structure of the complex (Baniulis et al., 2008; Kurisu et al., 2003; Malone et al., 2019; Stroebel et al., 2003). Based on modeling studies employing the *Orientation of Proteins in Membranes* (OPM) software (Lomize et al., 2006), it has been suggested that the cyt *b_6_f* complex protrudes ~2 nm from the lipid bilayer surface into stromal surface (Kirchhoff et al., 2017), that is, small changes in the stroma gap in grana as reported here (~0.6 nm) for light‐treated samples could be sufficient to restrict access to stacked grana. However, this hypothesis must be tested experimentally.

The factors involved in changes in the thylakoid architecture are not fully known. Recent studies have illuminated three groups of proteins that may control the thylakoid architecture: thylakoid‐localized ion transporter/channels (Finazzi et al., 2015), phosphatases and kinases (STN7 and STN8 kinases) (Fristedt & Vener, 2011; Fristedt et al., 2009; Wunder et al., 2013), or structural proteins like CURT1 localized in the grana margins (Armbruster et al., 2013; Puthiyaveetil et al., 2014) or RIQ (Yokoyama et al., 2016) that control thylakoid ultrastructure (see also Rantala et al. (2020)). The lumenal width could increase by osmotic water influx due to light‐driven acidification of the lumen, which may cause proton motive force‐driven influx of ions. Among the thylakoid ion transporters/channel proteins, some of the potential candidates that may be involved are *K^+^‐Efflux Antiporter 3 (*KEA3 (Kunz et al., 2014; Wang et al., 2017), *Voltage gated Chloride Channel 1* (VCCN1 (Herdean, et al., 2016a)), and a further chloride channel ClCe (Herdean, et al., 2016b; Marmagne et al., 2007).

An interesting detail of this study is the observed thinning of the thylakoid membrane in the light corroborating previous reports (Johnson et al., 2011; Kirchhoff et al., 2011; Murakami & Packer, 1970) This light‐induced membrane thinning is discussed to be of high relevance for the activation of photoprotective high‐energy quenching by light harvesting complexes (LHCs) (Ruban, 2019) and for protein import into the lumen from the stroma across the thylakoid membrane by the so‐called twin‐arginine translocation (TAT) pathway (McNeilage, 2016; New et al., 2018). The membrane thicknesses derived here (5.1 nm for dark‐ and 4.8 nm for light‐adapted plants) are higher than the expected dimension for the thylakoid membrane. Neutron and x‐ray scattering data for thylakoid membranes on leaves range from 3.4 to 4.8 nm (Jakubauskas et al., 2019). However, the light‐induced shrinkage in membrane thickness derived in this study is statistically highly significant indicating that thinning of thylakoid membranes could control essential membrane functions.

**FIGURE 9:**
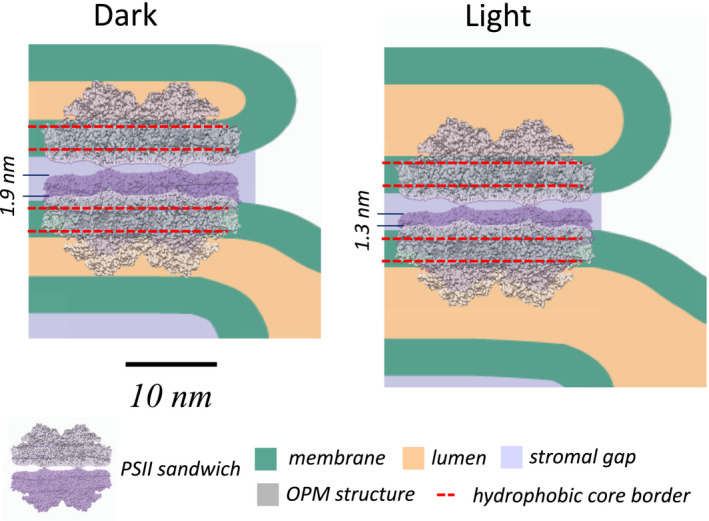
In‐scale model of the vertical organization of two PSII supercomplexes located in adjacent grana membranes for dark‐ and light‐adapted plants. PSII was placed into the (green) thylakoid membrane by using OPM modeling (the red dashed lines indicate the position of the hydrophobic lipid bilayer region). The ‘PSII sandwich” model was adapted from Albanese et al., 2017. The modeling shows that two adjacent PSII must be separated farther across the stroma gap in native grana compared to the isolated PSII sandwich (indicated by the numbers on the left). For further details, see in the text

Recently, the vertical organization of PSII‐LHCII supercomplexes localized in adjacent grana membranes comes into focus (e.g., Albanese et al., 2017, 2020; Dekker & Boekema, 2005; Wietrzynski et al., 2020). An important question is whether short‐range hydrogen bonds across the stroma gap between distinct amino acids on opposite PSII‐LHCII complexes could mediate grana stacking. These bonds were recently identified (Albanese et al., 2017, 2020) by elegant mass spectroscopic studies combined with cross‐linking on isolated dimers of PSII‐LHCII supercomplexes (PSII sandwich, see legend Figure 9). However, modeling the vertical organization of two PSII‐LHCII supercomplexes in our quantitative thylakoid model (Figure 8) reveals that for both dark‐ and light‐adapted states two adjacent PSII supercomplexes must be farther separated in native grana (Figure 9). From Figure 9, it can be estimated that the closest approximation of PSII parts on the stroma side is not smaller than ~1.5 nm making hydrogen‐bond–mediated membrane stacking (effective in sub‐nm range) unlikely and favors delocalized stacking models based on the balance of diffuse physicochemical forces (Puthiyaveetil et al., 2017; Wietrzynski et al., 2020). We want to point out that the stroma gap measured in our study is in accordance with published numbers (Daum et al., 2010; Kirchhoff et al., 2011; Wietrzynski et al., 2020). Another function determined by the stroma gap is vertical intermembrane excitonic energy transfer between chlorophyll molecules bound to PSII and LHCII localized in adjacent grana membranes. It turns out that the stroma gap of ~4 nm is too large to allow intermembrane energy transfer between adjacent grana membranes, favoring intramembrane lateral excitation energy processes (Farooq et al., 2016; Kirchhoff et al., 2004; Lambrev et al., 2011).

## 5 CONCLUSIONS

To understand how physiological functions in thylakoid membranes depend on changes in the complex network of grana and stroma lamella robust ultrastructure information is required. We presented a methodical pipeline for adequate sample preparation of leaf discs, over image quality check to computer‐based image analysis to quantify critical parameters of thylakoid membranes based on TEM images. Our results are consistent with expected changes in the lumen and stromal gap in thylakoids. This procedure allows to analyze physiologically relevant changes in the lumen and stromal gap under different light conditions from preselected images within a short period. We demonstrated that in the presence of light, lumenal swelling occurs both in the stacked and the unstacked regions of thylakoids. Furthermore, light induces small but significant decrease in the stroma gap in grana, and of the thylakoid membrane. Finally, our quantitative thylakoid modeling makes both hydrogen bond‐mediated grana stacking as well as vertical intermembrane energy transfer unlikely. In the future, our method can be used to further study the thylakoid architecture from different kinds of wild‐type and mutant plant tissue and altering environmental conditions leading to structural understanding as a foundation for the multiple functions associated with photosynthetic energy conversion.

## CONFLICT OF INTEREST

The authors declare no conflict of interest.

## AUTHOR CONTRIBUTIONS

M.L., R. M., H.M.O.O, V.S., and D.L.M. performed experiments and analyzed data. M.L., R. M., H.M.O.O, V.S., D.L.M, and H.K. designed the study, and H.K wrote the manuscript.

## Supporting information

Fig S1‐S3‐Table S1‐S2
