# Supplementary material for: Measuring the dynamic response of the thylakoid architecture in plant leaves by electron microscopy
Source: Plant Direct. 2020 Nov 5;4(11):e00280. doi: 10.1002/pld3.280 (PMC7644818; doi:10.1002/pld3.280)
Supplement: Supplementary file 1 — Fig S1‐S3‐Table S1‐S2 [file PLD3-4-e00280-s001.pdf]

SUPPLEMENTARY INFORMATION:

Supplementary Methodology:

Methods to analyze TEM images: Following are the two methods to analyze the TEM images

Method 1- The TEM images of grana stacks were analyzed by using ImageJ (Rueden et al. 2017) and a customized Python script. The general workflow and principle are as described in SI Fig. 1. First, the grayscale profile is plotted by using ImageJ "Plot profile" function. The generated "x, y" data were then analyzed by the customized Python code, ThylakoidVD_csv.py, which is available at https://github.com/limengwsu/Thylakoid_VD

The analysis was done by the following steps. Assuming the darkest stained parts of a grana are stroma gaps. For one granum repeat (or one thylakoid grana disc), the script first identifies the lowest grayscale value (dark valley), then scans the two secondary peaks (shoulders) of on two sides of the dark valley. These shoulders and dark valley grayscale values are normalized in the scale of 0~1 on each side, then the normalized values with x-axis values (distances) were fit to the Gaussian Function:

$$y = 1 - \frac{(x - \mu)^2}{2\sigma^2}$$

The calculated μ is used as a stroma gap center, while the σ is used to calculate stroma gap width, using the full width at half maximum, i.e. stroma gap width (S_G):

$$S_G = 2.355 \times \sigma$$

By repeating above calculations for a grana stack, the following series of numbers are expected:

Stroma gap centers: μ_1, μ_2, μ_3, … and S_{G1}, S_{G2}, S_{G3}, …

Then repeat distances (R_d) can be calculated as:

$$R_{d1} = \mu_2 - \mu_1$$

or: $R_{di} = \mu_{i+1} - \mu_i$, where i = 1, 2, 3, …

The "membranes plus lumen thickness" (M_L) can be calculated as:

$$M_{Li} = R_{di} - \frac{S_{Gi} + S_{Gi+1}}{2}$$

Along the calculation, original data and fit data were plotted and exported as pdf files for inspections later to remove bad fits and miscalculations, which usually are expected in poorly contrasted sample images. In our analysis by Method 1, numbers generated with the S_G < 2 nm, or R_d <13 nm were removed.

Method 2- The images are then cropped. The smooth function is applied to the pixels of the image and then the threshold is applied. This converts the image into arrays of gray values of '0' (corresponding to black) and '255' (corresponding to white). The borders between the individual black and white stripes are then extracted using a script in R program[1]. The border values are plotted with the plot dimensions being equal to the pixel dimensions of the image. The plot of the borders is transposed on the real image. Figure 2 explains the work-flow for image processing. On a good match of the real image and plot of borders the extracted values are used to

obtain lumen with membrane distance, stromal gap distance. This is done by multiplying the distance between stripes to pixel/nm of the image. Repeat distance is the sum calculated from half of one lumen distance to the next one, or half of stromal gap distance to the consecutive one respectively. The repeat distance calculations are re-verified using a separate logic of calculation from the raw image using Python script (as described in Method 1).

Supplementary Figures:

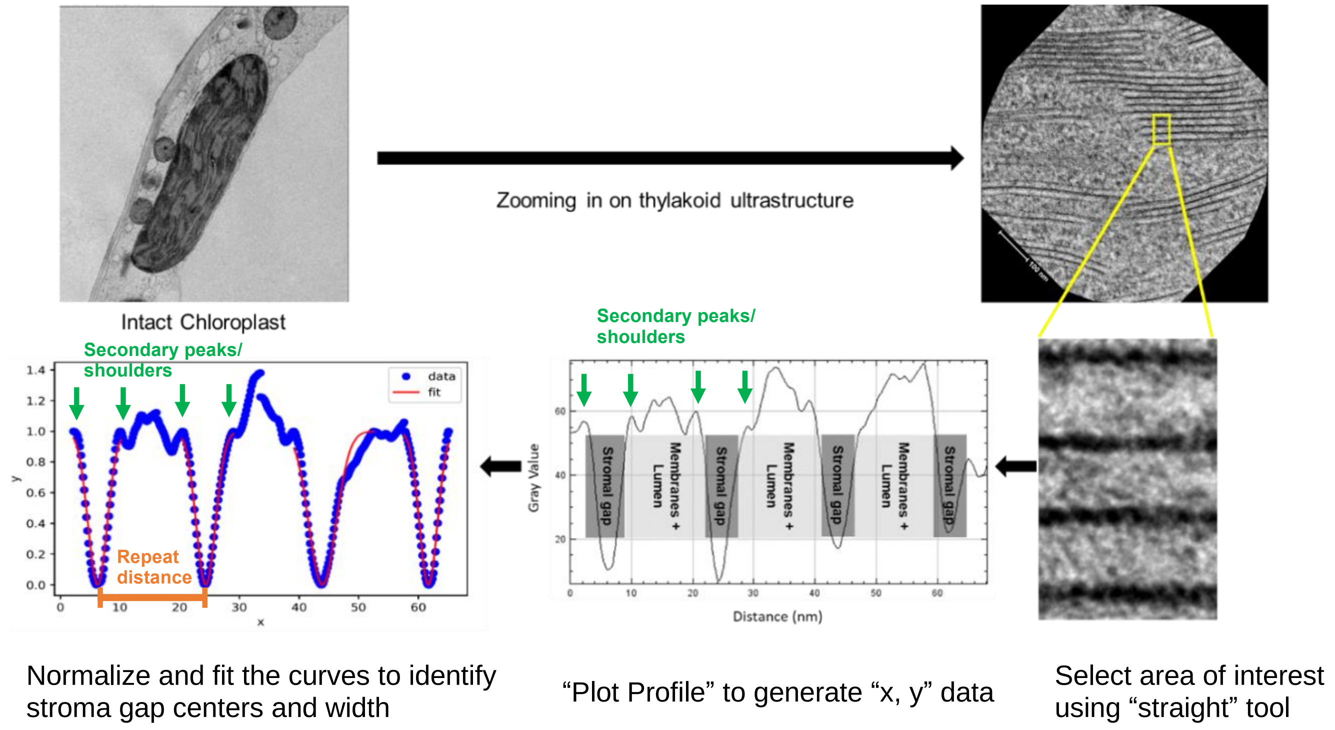

SI Figure 1. Flow diagram of Method 1. Green arrows point at "shoulders" identified and used for calculating the stroma gap. Orange bars indicate how repeat distance is defined and calculated. See text and script deposit for details.

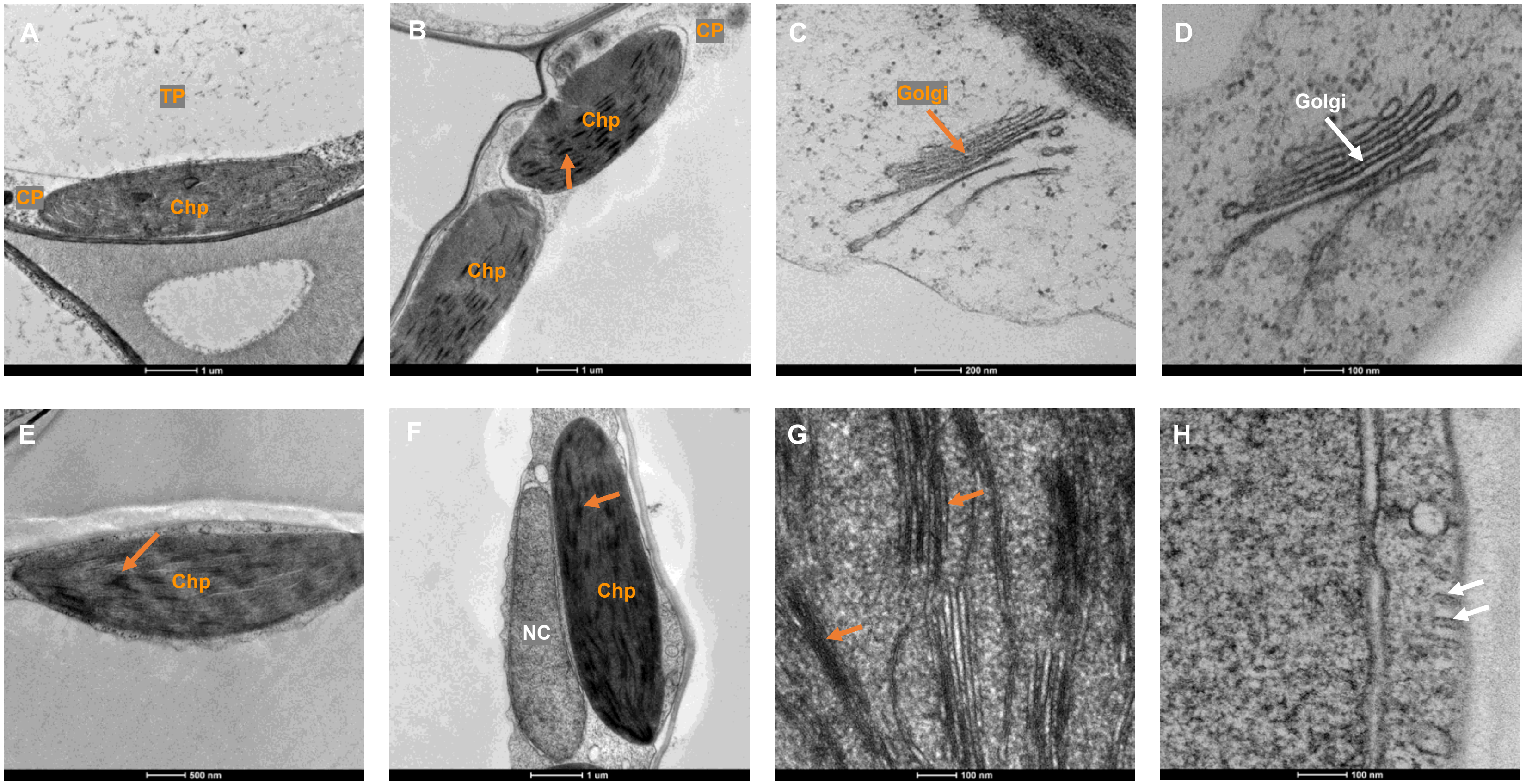

SI Figure 2. Examples of samples with different quality. Orange characters and arrows point at damaged structures. A-B, examples of broken chloroplasts and other structures. C-D, comparison of Golgi apparatus. E-G, compressed thylakoids. H, example of other organelle/structures: the two arrows point to microtubules. Nucleus (NC), plasma membrane (PM), cytoplasm (CP), chloroplast (Chp), tonoplast (TP) are denoted.

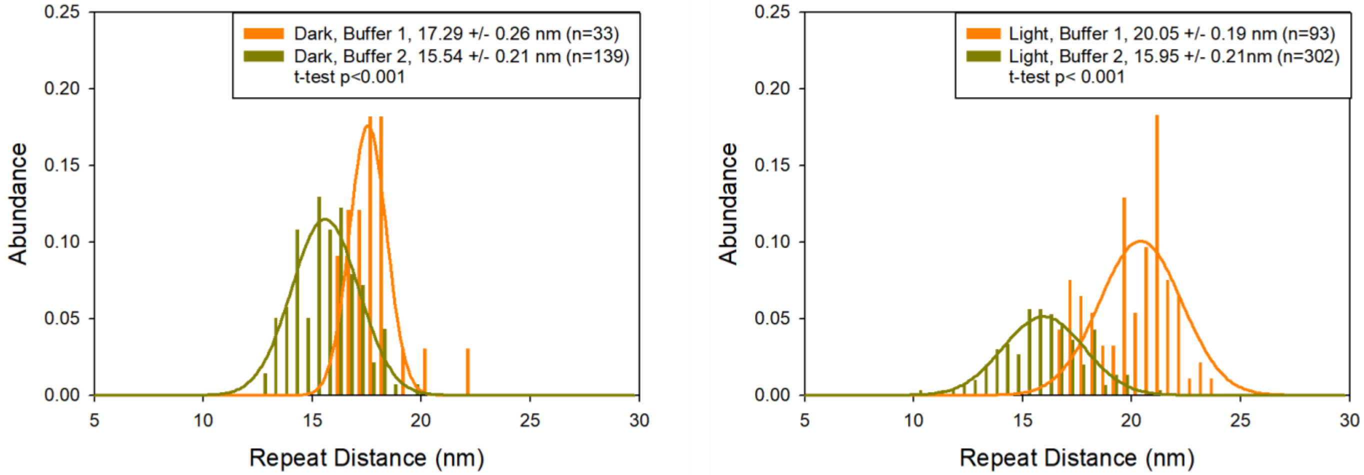

SI Figure 3. Comparison between the two microwave fixed samples from light and dark-adapted thylakoids.

SI Table 1. Experiments attempted for studying Arabidopsis thylakoid ultrastructure using HPF-FS.

No. of leaf discs tested	D/L[a]	Cryo-protectant	Cryo-filler	Infiltration	No. of intact leaf structure	Overall cell structure	Plasmolysis	Cytoplasm	Tonoplast	Chloroplast
12	D&L	50 MES pH 6.5 + 20% BSA +Enz.[b]	Cryo-protectant	Vacuum	0	Broken	No	Damaged	Broken	Mostly broken
12	D&L	15% Dextran in 50 mM Tricine pH 7.4	Cryo-protectant	Vacuum	0	Broken	Some	Damaged	Broken	Mostly broken
12	D&L	50 mM Tricine pH 7.4 15% Dextran + 10% Methanol	Cryo-protectant	Vacuum	0	Broken	Some	Damaged	Broken	Mostly broken
6	D	0.33 M sorbitol	Yeast paste [c]	Syringe	0	Some intact	Some	Okay	Some okay	Okay
*6**	D	0.33 M sorbitol	Yeast paste	Syringe	0	Broken	Some	Damaged	Broken	Mostly broken
12	D&L	315 mM sorbitol +Enz. + 1mM $NaHCO_3$	Cryo-protectant	Syringe	0	Some intact	Some	Some Damaged	Some okay	Some damaged
6	D	None	Yeast paste	None	0	Some intact	N.A. [d]			
6	D	300 mM Sorbitol + 10% methanol	Cryo-protectant	Syringe	3 almost intact	Mostly intact †	No	Mostly okay	Some okay	Mostly okay
6	D	300 mM Sorbitol	Cryo-protectant	Syringe	2 almost intact	Some intact	No	Mostly okay	Some okay	Mostly okay
6	D	300 mM sorbitol + Enz. + 1mM $NaHCO_3$	Cryo-protectant	Syringe	3 almost intact	Some intact	No	Some okay	Some okay	Some okay
6	D	300 mM Sorbitol + 10% methanol	Cryo-protectant	Syringe	6 almost intact	Mostly intact	No	Mostly okay	Some okay	Some okay
6	D	300 mM sorbitol + Enz. + 1mM $NaHCO_3$	Cryo-protectant	Syringe	2 almost intact	Some intact	No	Mostly damaged	Mostly damaged	Mostly damaged
12	D&L	300 mM sorbitol + Enz. + 1mM $NaHCO_3$	300 mM Sorbitol + 10% methanol	Syringe	6 almost intact	Mostly intact	No	Mostly okay	Some okay	Some okay
6	D	300 mM Sorbitol + 10% methanol	Cryo-protectant	Syringe	3 almost intact	Mostly intact	No	Mostly okay	Some okay	Mostly damaged
6	D	300 mM sorbitol + Enz. + 1mM $NaHCO_3$	300 mM Sorbitol + 10% methanol	Syringe	1 almost intact	Mostly intact	No	Mostly damaged	Mostly damaged	Mostly damaged
12	D	300 mM Sorbitol + 10% methanol	Cryo-protectant	Syringe	6 almost intact	Mostly intact	No	Mostly okay	Some okay	Mostly okay
12	D	300 mM Sorbitol + 10% methanol	Cryo-protectant [e]	Syringe	9 almost intact	Mostly intact	No	Mostly okay	Some okay	Mostly okay
9	L	300 mM Sorbitol + 10% methanol	Cryo-protectant [e]	Syringe	7 almost intact	Mostly intact	No	Mostly okay	Some okay	Mostly okay

[a] D/L indicates samples are dark adapted (D) or light treated (L). The light treated leaf discs were loaded in carriers with sapphire discs for experiments before Jan-2017.
[b] Enz. indicates enzyme mix (5mM glucose +10U/mL gluc oxidase + 100U/mL catalase), added to avoid oxygen gas bubble.
[c] Yeast paste is made with 10% methanol by mixing equal volume of powder and liquid, adapted from Bobik et al. 2014.
[d] not available, no TEM images were taken due to excessive damage.
[e] these carriers were pre-coated with hexadecane instead of lecithin.
** kea3-1 mutant*
† Note that the "mostly intact/okay" notation does NOT mean that all samples passed "leaf structure" quality have the same intactness at cellular or sub-cellular level. The notation is given if **at least one** sample has such feature of "mostly intact/okay".

SI Table 2. Variation in thylakoid architecture in HPF-FS leaf samples prepared in different experiments, analyzed using Method 1.

Preparation No.	Leaf treatment	Cryoprotectant	No. of leaf discs (sample blocks) analyzed	Sample size † (layers of thylakoid repeats)	Lumen + membranes (nm, mean ± s.e.m)	Stroma gap (nm, mean ± s.e.m)	Repeat distance (nm, mean ± s.e.m)
1	Dark adapted	300 mM Sorbitol + 10% methanol	1	330	13.59 ± 0.12	3.69 ± 0.05	17.31 ± 0.11
2	Dark adapted	300 mM Sorbitol	1	255	14.40 ± 0.14	3.68 ± 0.05	18.11 ± 0.14
3	Dark adapted	300 mM sorbitol (anaerobic)§	1	197*	11.90 ± 0.12	3.38 ± 0.04	15.30 ± 0.12
4	Dark adapted	300 mM Sorbitol + 10% methanol	2	112	12.30 ± 0.25	5.05 ± 0.12	17.39 ± 0.23
5	Dark adapted	300 mM Sorbitol + 10% methanol	1	495	14.34 ± 0.12	3.72 ± 0.05	18.07 ± 0.11
6	Dark adapted	300 mM Sorbitol + 10% methanol	2	1080	13.21 ± 0.06	3.90 ± 0.03	17.09 ± 0.06
7	Light adapted	300 mM Sorbitol + 10% methanol	2	1080	16.33 ± 0.10	3.55 ± 0.03	19.89 ± 0.10

1. **Rueden CT, Schindelin J, Hiner MC, DeZonia BE, Walter AE, Arena ET, Eliceiri KW** (2017) ImageJ2: ImageJ for the next generation of scientific image data. BMC bioinformatics **18:** 529
2. **Chambers, J**. Software for data analysis: programming with R. Springer Science & Business Media: **2008**.
3. **Bobik, K., Dunlap, J.R. and Burch-Smith, T.M.,** 2014. Tandem high-pressure freezing and quick freeze substitution of plant tissues for transmission electron microscopy. *JoVE (Journal of Visualized Experiments)*, (92), p.e51844.
